# Long-Term Visit-To-Visit Blood Pressure Variability and Cognition: A Systematic Review of Observational Studies

**DOI:** 10.1093/geroni/igab046.2609

**Published:** 2021-12-17

**Authors:** Tomiko Yoneda, Jamie Knight, Amelia Zhang, Graciela Muniz-Terrera, Andrea Piccinin

**Affiliations:** 1 University of Victoria, Victoria, British Columbia, Canada; 2 University of Toronto, Toronto, Ontario, Canada; 3 University of Edinburgh, Edinburgh, Scotland, United Kingdom

## Abstract

Existing literature suggests that in comparison to a single blood pressure (BP) measurement, or the mean of multiple recordings, BP variability (BPV) may reflect dysfunction in cardiovascular regulatory mechanisms, leading to compromised cognitive health. No systematic review has yet synthesized observational reports examining the association between cognition and long-term visit-to-visit BPV. In response, a comprehensive literature search was executed in December, 2019, and updated in December, 2020. Methodological approach was pre-registered (https://osf.io/vmnuq/). Of 1385 reports, 27 met eligibility criteria. Most executed secondary analyses using existing longitudinal datasets of older adults (N=21). Intervals between measurement occasions ranged from 30 days to four years, and follow-up ranged from 0.5-25 years. Most studies computed more than one index of BPV (range=1-6), and all included at least three BP recordings (range=3-12). Given extensive between-study variability in analytic approach (e.g., BPV and cognition treated as continuous and/or categorical variables; number of covariates ranged 0-18), our team determined that meta-analyzing the results would be inappropriate. Despite heterogeneity in study characteristics, the majority (85.2%) reported that systolic BPV (sBPV) was negatively associated with cognition; specifically, higher sBPV was associated with cognitive impairment (N=9), cognitive decline (N=6), and/or risk of dementia (N=5). Four studies also revealed higher sBPV in individuals with dementia compared to controls. Three studies reported no association, while one reported a positive significant association between BPV and cognition. Results were similar for diastolic BPV. Despite considerable heterogeneity in study characteristics, greater variability in visit-to-visit BP appears to be consistently associated with adverse cognitive outcomes.

